# Pepsin Promotes Activation of Epidermal Growth Factor Receptor and Downstream Oncogenic Pathways, at Slightly Acidic and Neutral pH, in Exposed Hypopharyngeal Cells

**DOI:** 10.3390/ijms22084275

**Published:** 2021-04-20

**Authors:** Panagiotis G. Doukas, Dimitra P. Vageli, Clarence T. Sasaki, Benjamin L. Judson

**Affiliations:** The Yale Larynx Laboratory, Department of Surgery (Otolaryngology), Yale School of Medicine, New Haven, CT 06510, USA; panagiotis.doukas@yale.edu (P.G.D.); clarance.sasaki@yale.edu (C.T.S.); benjamin.judson@yale.edu (B.L.J.)

**Keywords:** pepsin, EGFR, STAT3, NF-*κ*B, laryngopharyngeal reflux, non-acidic reflux, pH, hypopharyngeal primary cells, hypopharyngeal cancer, head and neck cancer

## Abstract

Pepsin refluxate is considered a risk factor for laryngopharyngeal carcinogenesis. Non-acidic pepsin was previously linked to an inflammatory and tumorigenic effect on laryngopharyngeal cells in vitro. Yet there is no clear evidence of the pepsin-effect on a specific oncogenic pathway and the importance of pH in this process. We hypothesized that less acidic pepsin triggers the activation of a specific oncogenic factor and related-signalling pathway. To explore the pepsin-effect in vitro, we performed intermittent exposure of 15 min, once per day, for a 5-day period, of human hypopharyngeal primary cells (HCs) to pepsin (1 mg/mL), at a weakly acidic pH of 5.0, a slightly acidic pH of 6.0, and a neutral pH of 7.0. We have documented that the extracellular environment at pH 6.0, and particularly pH 7.0, vs. pH 5.0, promotes the pepsin-effect on HCs, causing increased internalized pepsin and cell viability, a pronounced activation of EGFR accompanied by NF-*κ*B and STAT3 activation, and a significant upregulation of *EGFR*, *AKT1*, *mTOR*, *IL1β*, *TNF-α*, *RELA(p65)*, *BCL-2*, *IL6* and *STAT3*. We herein provide new evidence of the pepsin-effect on oncogenic EGFR activation and its related-signaling pathway at neutral and slightly acidic pH in HCs, opening a window to further explore the prevention and therapeutic approach of laryngopharyngeal reflux disease.

## 1. Introduction

Laryngopharyngeal reflux (LPR) is a widely common disease that affects nearly 10% of the U.S. population [1]. It is associated with gastric and duodenal refluxate to the supra-esophageal upper and respiratory tract [2]. Due to the refluxate components, LPR is considered to be a risk factor for chronic inflammatory disorders and head and neck squamous cell carcinoma (HNSCC) [3,4]. One of the gastric components that, interestingly, may play a role in this inflammatory and carcinogenic process is pepsin [5,6].

Pepsin is a protease that is physiologically present in gastric juice and is one of the components of refluxate that reaches the larynx, at >210 ng/mL [7,8,9,10,11], and at variable pH, including strongly acidic (pH < 4.0), acidic (pH 4.0–5.0), as well as less acidic pH (pH > 5.0) [11,12,13,14,15,16,17,18]. Pepsin is primarily active at strongly acidic pH < 4.0, and so at weakly acidic pH 5.0–6.0 remains less active. Johnston et al., have shown complete inactivation of pepsin at pH 6.5 and that pepsin at pH 7.0, although inactive, remains stable for at least 24 h at 37 °C [19].

The LPR episode occurs when refluxate fluid at pH < 4.0 reaches the upper aerodigestive tract, and usually manifests as hoarseness and cough. Current therapy mainly targets the neutralization of the refluxate’s pH that may improve symptoms and prevent acidic pepsin-containing refluxate harmful effects [2,20]. However, several clinical studies have shown measurements of less acidic pH (4.0–7.0) in patients with gastroesophageal reflux disease or LPR, supporting a harmful effect of less acidic refluxate containing pepsin in asymptomatic patients [12,13,14,15,16,17,18]. Oelschlager et al., for example, showed that the majority of reflux episodes that reached the pharynx were non-acidic, and thus did not cause symptoms [12]. Kawamura et al., using a pH reflux catheter, showed that gastric reflux at a less acidic pH (>4.0) was common in patients with LPR-related inflammatory disease [13]. Formánek et al., monitoring pH for 24 h in patients with laryngeal pathology, showed a significantly higher number of patients with pepsin-positive reflux episodes at less acidic pH (4.0–7.0), compared with patients who had pepsin-negative episodes [14]. Formánek et al. also showed in this study that episodes of reflux at pH above 7.0, either positive or negative for pepsin, were rare. It is clear that pH is a critical factor in pepsin-induced hypopharyngeal pathology.

Acidic pepsin (pH < 4.0) can dramatically affect the cell viability of laryngopharyngeal cells [21,22], while neutral pepsin (pH 7.0) can promote the expression of cell hyperproliferation markers [6,21,23]. The proposed mechanism by which pepsin can exert its tumorigenic effect at non-acidic pH, in laryngopharyngeal epithelial cells, is proven to be through its internalization by receptor-mediated endocytosis [6,21,24]. In 2009, Samuels and Johnston presented evidence that pepsin in a non-acidic environment can lead to the production of inflammatory cytokines [25]. Additionally, work by Johnston et al. showed that pepsin is taken up by hypopharyngeal epithelial cells, inducing expression of genes related to stress and toxicity at neutral pH [21,24,26]. Also, recent data from a gene enrichment analysis supported that single exposure to neutral pepsin can simulate the activation of signaling pathways related to cancer and inflammation [27]. Yet there is no evidence that a specific oncogenic pathway, nor key molecules, have been linked to downstream antiapoptotic pathways in HNSCC, activated by pepsin at less acidic or neutral pH in this process.

HNSCC is characterized by the upregulation of multiple and complex signaling pathways that have attracted interest in prevention and target therapy. Common oncogenic factors and their related antiapoptotic pathways involved in head and neck oncogenesis include EGFR/Akt/PI3K/mTOR, EGFR/Ras/RAF/MAPK, TNF/ΙΚΚ/NF-κB, IL6/STAT3, wnt/β-catenin, etc. [28,29,30,31,32,33,34]. Several cytokines such as IL-1β, and IL-6 have also been proposed to play a key role in inflammatory-related carcinogenesis in HNSCC [35,36].

Epidermal growth factor receptor (EGFR) is widely known to be commonly over-expressed in many epithelial cancers, including 80–100% of HNSCC [37,38]. EGFR is a transmembrane glycoprotein and is activated through the binding of epidermal growth factor and other ligands. After its activation, receptor dimerization, and autophosphorylation, EGFR stimulates a downstream of several oncogenic signaling pathways such as AKT, mTOR, NF-*κ*B, and STAT3 [39,40,41,42,43,44]. Van Waes’s team has also demonstrated EGFR overexpression in the HNSCC cell line and suggested that EGFR signaling activates NF-*κ*B related pathways and downstream genes [45]. Van Waes’s and other teams have also revealed a crosstalk between NF-κB and other classical downstream pathways of EGFR, such as AKT and STAT3, to promote gene expression [46,47,48].

Nuclear factor kappa B (NF-*κ*B) is a known key molecule between inflammatory and carcinogenetic processes [49,50]. NF-*κ*B is often upregulated in HNSCC [51] and promotes the upregulation of downstream anti-apoptotic factors, such as *BCL2* [52], *STAT3* [46], *TNF-α* [32,53], and various others cytokines related to oncogenesis, like *IL6* and *IL1β* [35,36]. NF-κB activation has also been associated with PI3K/AKT/mTOR oncogenic pathway, in head and neck cancer [47,48]. The underlying mechanistic role of an NF-*κ*B-related antiapoptotic pathway specific to LPR-related oncogenesis has been recently documented by our in vitro and in vivo models [54,55,56,57,58,59,60].

Here, we attempt to explore whether pepsin can activate oncogenic factors such as EGFR, NF-*κ*B and STAT3 and upregulate their related oncogenic pathways in human hypopharyngeal primary cells, and the role of the weakly acidic pH 5.0, slightly acidic pH 6.0 and neutral pH 7.0 in this pepsin-effect. Understanding the pepsin-effect and its related oncogenic pathways and key molecules may contribute to the developing of new preventive and/or therapeutic approach in LPR disease.

## 2. Results

### 2.1. Slightly Acidic and Neutral Pepsin Preserves Cell Viability in HCs

We performed a cell viability assay to determine if exposure to pepsin at a weakly acidic pH of 5.0, slightly acidic pH of 6.0, or neutral pH of 7.0 affects the survival of HCs.

**Figure 1:**
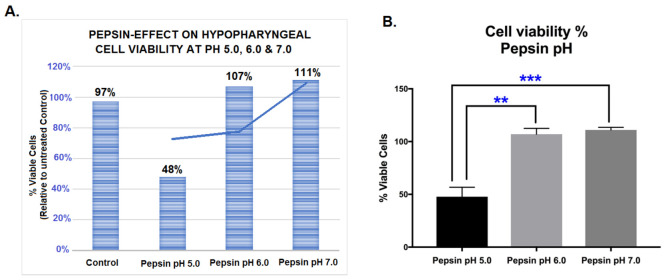
The pepsin-effect on cell viability of human hypopharyngeal primary cells (HCs) exposed to weakly acidic pH 5.0, slightly acidic pH 6.0 and neutral pH 7.0. (**A**). Graphs depict the viability rates in HCs exposed to control (media at pH 7.0) and pepsin at pH 5.0, 6.0 and pH 7.0 (% of viable HCs in pepsin and control-treated groups vs. untreated control). (**B**). Graph depicts differences in the viability of HCs treated with pepsin at pH 5.0, 6.0 and 7.0 (pepsin treated vs. control-treated HCs). Graphs, created by GraphPad Prism 7 software; ** *p* < 0.005; *** *p* < 0.0005, by *t*-test; multiple comparisons by Holm-Sidak). (Data are derived from three independent experiments).

The results of our assay revealed that cell viability was preserved with both slightly acidic and neutral pepsin, at pH 6.0 and 7.0, respectively (Figure 1A). Treatment with weakly acidic pepsin at pH 5.0 reduced cell viability of HCs compared to untreated controls (Figure 1A).

Specifically, HCs exposed to pepsin at pH 6.0 or 7.0 resulted in significantly higher percentages of viable cells compared to those exposed to pepsin at pH 5.0 (Figure 1B) (*p* < 0.05, multiple *t*-tests).

Due to pepsin being capable of undergoing endocytosis by exposed epithelial cells, to confirm whether exposure to pepsin at weakly acidic (pH 5.0), slightly acidic (pH 6.0), or neutral (pH 7.0) results in internalization by HCs, we performed immunofluorescence staining for pepsin A.

Immunofluorescence assay confirmed that pepsin at either pH 5.0, 6.0, or 7.0 is found intracellularly in the treated HCs (Supplementary Material; Figure S1A). Intracellular pepsin was significantly higher at pH 7.0 compared to pH 6.0 or 5.0 (*p* < 0.05; *t*-test) (Supplementary Material; Figure S1B).

### 2.2. Slightly Acidic and Neutral Pepsin Results in EGFR Activation and bcl-2 Overexpression in HCs

We performed immunofluorescence assay, ELISA and/or Western blot analysis for EGFR phosphorylated at Tyr1092 and bcl-2 in order to explore if exposure to weakly acidic (pH 5.0), slightly acidic (pH 6.0) or neutral (pH 7.0) pepsin can activate EGFR and induce bcl-2 overexpression in HCs.

**Figure 2:**
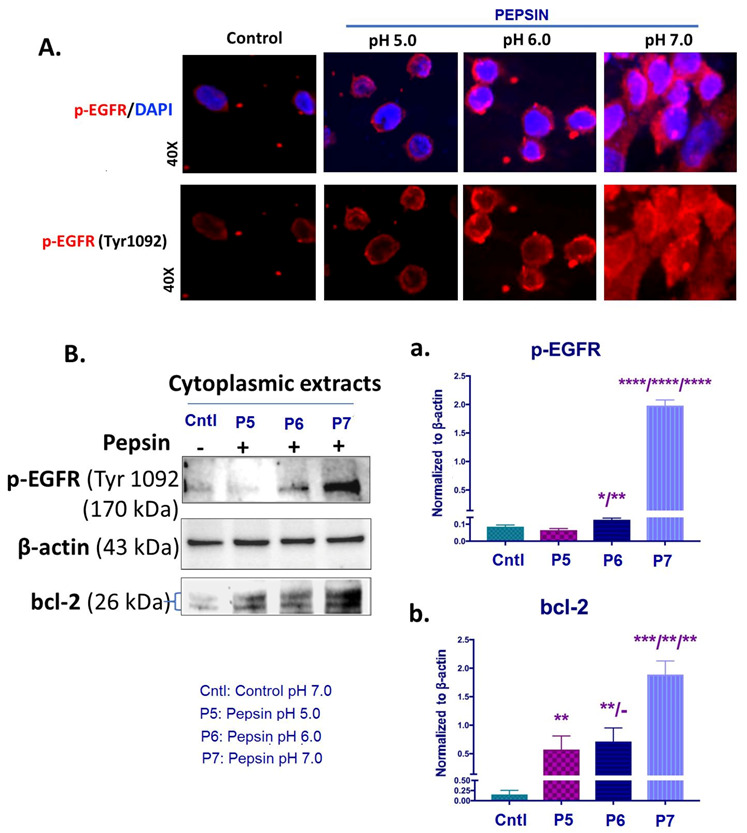
The pepsin-effect on EGFR activation and bcl-2 overexpression in HCs exposed to weakly acidic pH 5.0, slightly acidic pH 6.0 and neutral pH 7.0. (**A**) Immunofluorescence staining for p-EGFR (Tyr1092) (red: p-EGFR; blue: DAPI for nuclear DNA staining; scale bar 20 um by Zen imagining software). (**B**) Graphs depict the cytoplasmic protein levels of p-EGFR (Tyr1092) and bcl-2 (**a**,**b**) in pepsin and control-treated HCs, by western blot analysis (β-actin was used to normalize cytoplasmic protein extracts) (*t*-test; multiple comparisons by Holm-Sidak, * *p* < 0.05; ** *p* < 0.005; *** *p* < 0.0005; **** *p* < 0.00005; GraphPad Prism 7.0; means ± SD of three independent experiment).

Our analyses revealed that exposure to pepsin at pH 6.0 and pH 7.0 resulted in significant activation of EGFR, compared to treatment at pH 5.0 and controls (Figure 2A,B and Supplementary Material; Figure S2A).

Specifically, immunofluorescence assay revealed an intense membrane/cytoplasmic staining for p-EGFR (Tyr1092) of HCs exposed to pepsin at pH 6.0 and pH 7.0. HCs treated with pepsin at pH 5.0 and controls (neutral media at pH 7.0 without pepsin) showed less intense p-EGFR staining, as shown in Figure 2A.

Western blot analysis and ELISA confirmed the above immunocytochemical data. As shown in Figure 2B and Supplementary Material Figure S2A, HCs exposed to pepsin at pH 6.0 or pH 7.0 showed significantly higher cytoplasmic levels of p-EGFR (Tyr1092) compared to treatment with pepsin at pH 5.0 and controls. In particular, treatment with pepsin at pH 7.0 caused an increase in p-EGFR protein levels greater than pepsin at pH 6.0 (*p* < 0.00005).

Western blot analysis revealed that exposure to pepsin at pH 7.0 caused a pronounced bcl-2 overexpression, with a significant difference compared to pepsin at pH 6.0 or pH 5.0 (Figure 2B). Our analysis revealed that pepsin at pH 5.0 and pH 6.0 produced significantly higher cytoplasmic bcl-2 levels in treated-HCs compared to controls.

In conclusion, neutral (pH 7.0) pepsin caused the highest levels of EGFR activation and bcl-2 overexpression levels in treated-HCs. Slightly acidic (pH 6.0) pepsin induced activation of EGFR and enhanced bcl-2 levels, however it was found to be significantly less than pepsin at neutral pH (7.0). Although weakly acidic (pH 5.0) pepsin increased bcl-2 levels, it did not activate EGFR.

### 2.3. Slightly Acidic and Neutral Pepsin Enhances STAT3 Activation in HCs

To explore if exposure to weakly acidic (pH 5.0), slightly acidic (pH 6.0) or neutral (pH 7.0) pepsin activates STAT3 in HCs, we performed immunofluorescence assay, ELISA and Western blot analysis for STAT3 phosphorylated at Tyr705.

**Figure 3:**
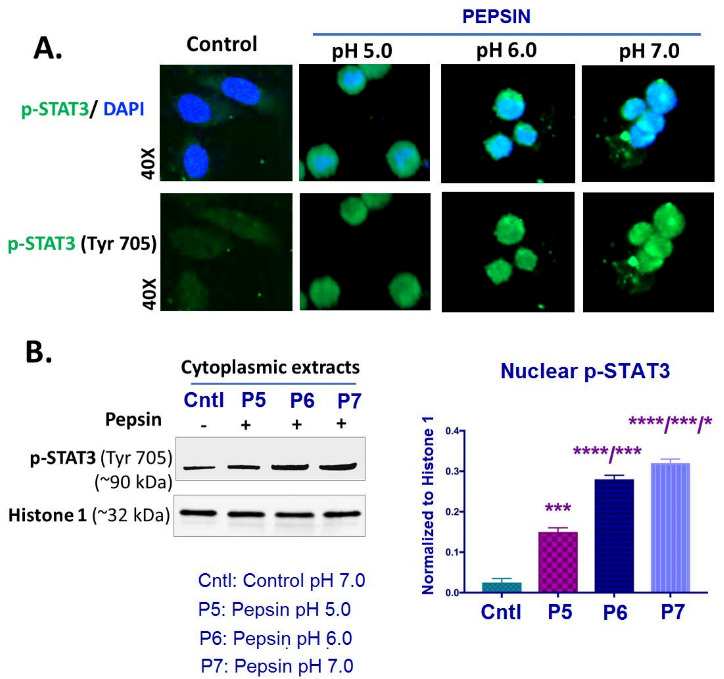
Effect of pepsin on STAT3 activation in HCs exposed at weakly acidic pH 5.0, slightly acidic pH 6.0 and neutral pH 7.0. (**A**) Immunofluorescence staining for p-STAT3 (Tyr705) (green: p-STAT3; blue: DAPI for nuclear DNA staining; scale bar 20 um by Zen imagining software). (**B**) Graph depicts the nuclear protein levels of p-STAT3 (Tyr705) in pepsin and control-treated HCs, by western blot analysis (Histone 1 was used to normalize nuclear protein extracts). (*t*-test; multiple comparisons by Holm-Sidak, * *p* < 0.05; *** *p* < 0.0005; **** *p* < 0.00005; GraphPad Prism 7.0; means ± SD of three independent experiment).

Exposure to pepsin at pH 6.0 and particularly at pH 7.0 caused an abundant STAT3 activation in treated HCs compared to controls (Figure 3A,B and Supplementary Material Figure S2B). Exposure to pepsin at pH 5.0 induced a less intense STAT3 activation.

**Figure 4:**
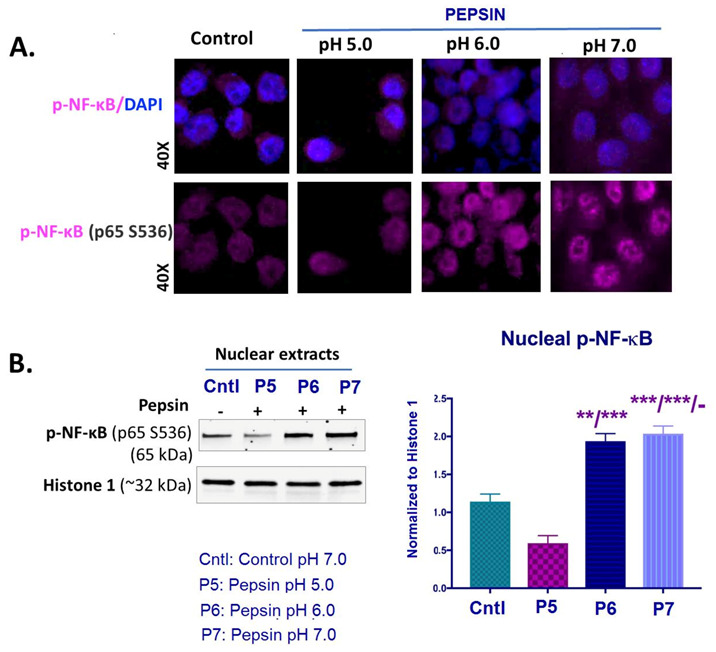
The effect of pepsin on NF-κB activation in HCs exposed at weakly acidic pH 5.0, slightly acidic pH 6.0 and neutral pH 7.0. (**A**)**.** Immunofluorescence staining for p-NF-κB (p65 S536) (purple: p-NF-κB; blue: DAPI for nuclear DNA staining; scale bar 20 um by Zen imagining software). (**B**). Graph depicts the nuclear protein levels of p-NF-κB (p65 S536) in pepsin and control-treated HCs, by western blot analysis (Histone 1 was used to normalize nuclear protein extracts). (*t*-test; multiple comparisons by Holm-Sidak, ** *p* < 0.005; *** *p* < 0.0005; GraphPad Prism 7.0; means ± SD of three independent experiment).

Specifically, immunofluorescence assay revealed an intense nuclear staining for p-STAT3 (Tyr705) of HCs exposed to pepsin at 6.0 and pH 7.0 (Figure 3A). HCs exposed to pepsin at pH 5.0 presented a less intense nuclear localization of p-STAT3 compared to pepsin at pH 6.0 or 7.0. Controls (HCs treated with neutral media at pH 7.0 without pepsin), as shown in Figure 4A, showed a very weak nuclear staining for p-STAT3.

Western blot analysis and ELISA confirmed the above immunocytochemical data. As shown in Figure 3B and Supplementary Material; Figure S2B, HCs exposed to pepsin at pH 6.0 and particularly at 7.0 produced significantly higher p-STAT3 (Tyr705) nuclear levels, compared to pepsin at pH 5.0 and controls (Figure 3B). HCs exposed to pepsin at pH 5.0 also presented higher nuclear levels of p-STAT3 compared to controls.

Summarizing, neutral (pH 7.0) pepsin caused the highest levels of STAT3 activation a in HCs, followed by slightly acidic (pH 6.0) pepsin. Although weakly acidic (5.0) pepsin increased p-STAT3 nuclear levels, this increase was significantly less than with slightly acidic and neutral pepsin.

### 2.4. Slightly Acidic and Neutral Pepsin Promotes NF-κB (p65) Activation in treated HCs

We performed an immunofluorescence assay, western blot analysis and ELISA for NF-κB (p65) phosphorylated at Ser536 in order to explore if exposure to weakly acidic (pH 5.0), slightly acidic (pH 6.0) or neutral (pH 7.0) pepsin can activate NF-κB in HCs. Our analyses revealed that exposure to pepsin at either pH 6.0 or 7.0 induced activation of NF-κB, in treated-HCs (Figure 4A,B).

Specifically, immunofluorescence assay revealed an intense nuclear staining for p-NF-κB (p65 S536) of HCs exposed to pepsin at pH 6.0 and pH 7.0 (Figure 4A). HCs treated with pepsin at pH 5.0 showed a less intense nuclear localization of p-NF-κB (p65 S536) compared to pepsin at pH 6.0 or 7.0 and controls. Controls (HCs treated with neutral media at pH 7.0 without pepsin), as shown in Figure 4A, showed a weak nuclear staining for p-NF-κB.

Western blot analysis confirmed the above immunocytochemical data. As shown in Figure 4B, HCs exposed to pepsin at pH 6.0 or 7.0 demonstrated significantly higher p-NF-κB (p65 S536) nuclear levels, compared to pepsin at pH 5.0 and controls (Figure 4B).

ELISA further showed that HCs exposed to pepsin ta pH 5.0 induced higher nuclear levels of total NF-κB (p65), compared to controls (Supplementary Material; Figure S2C). However, pepsin at pH 7.0 produced the highest nuclear levels of total NF-κB, with a significant difference compared to treatment at pH 6.0 or 5.0.

To summarize, slightly acidic (pH 6.0) or neutral (pH 7.0) pepsin promoted activation of NF-κB in treated HCs.

### 2.5. Pepsin Induces Elevated NF-κB Transcriptional Activity in Treated HCs, at Slightly Acidic pH 6.0 and Neutral pH 7.0

We performed luciferase assay in order to measure NF-*κ*B transcriptional activity in HCs under pepsin exposure at each pH point compared to control.

**Figure 5:**
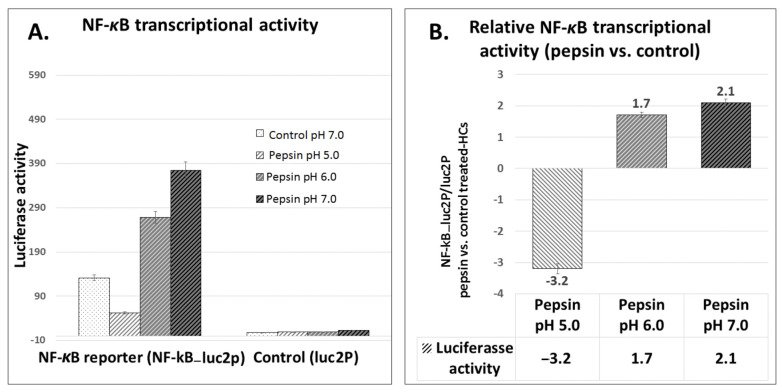
The effect of pepsin on NF-*κ*B transcriptional activity in HCs exposed at weakly acidic pH 5.0, slightly acidic pH 6.0 and neutral pH 7.0. (**A**) Columns represent luciferase NF-κB transcriptional activity (mean ± SD of two independent experiments) in HCs transfected with NF-*κ*B luciferase responsive element (NF-*κ*B_Luc2P) and control luciferase reporter (luc2P). (**B**) Columns represent ratios of relative luciferase NF-*κ*B transcriptional activity between pepsin treated groups and control (NF-*κ*B_Luc2P/Luc2P: NF-*κ*B luciferase responsive element/control luciferase reporter in pepsin vs. control treated-HCs).

The luciferase assay revealed that pepsin at slightly acidic pH 6.0 and particularly at neutral pH 7.0 increased NF-*κ*B transcriptional activity (NF-*κ*B_Luc2P) in HCs (Figure 5A). In contrast, the luciferase assay showed that HCs exposed to pepsin at weakly acidic pH 5.0 induced lower levels of NF-*κ*B transcriptional activity relative to controls.

Figure 5B shows the relative NF-*κ*B transcriptional activity in pepsin treated groups vs. controls. HCs exposed to pepsin at pH 6.0 and 7.0 demonstrated an increase of NF-*κ*B luciferase activity compared to controls. HCs treated with pepsin at pH 5.0 showed a decrease of NF-*κ*B of luciferase activity compared to controls.

### 2.6. Slightly Acidic and Neutral Pepsin Promotes the Transcriptional Activation of EGFR-Related Oncogenic Factors in HCs

We performed gene expression analysis by qPCR in order to detect if exposure to pepsin at weakly acidic (pH 5.0), slightly acidic (pH 6.0) or neutral (pH 7.0) conditions can induce a transcriptional activation of *EGFR* and its related signaling that has been associated with oncogenesis of HNSCCs.

**Figure 6:**
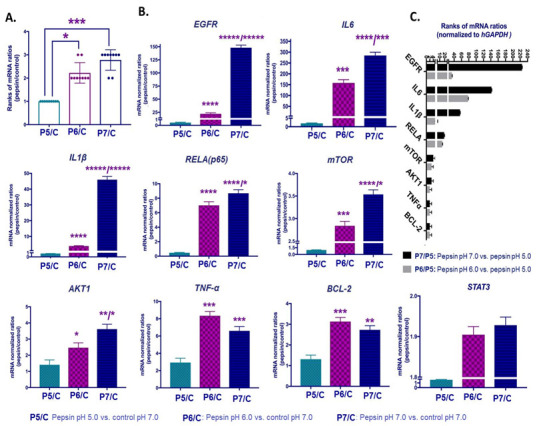
The pepsin-effect on transcriptional activation of EGFR-related signaling pathway in HCs exposed to weakly acidic pH 5.0, slightly acidic pH 6.0 and neutral pH 7.0. (**A**). Columns represent mRNA ratios of pepsin at pH 5.0, 6.0 and 7.0 vs. control treated-groups, by qPCR. (**B**) Columns represent mRNA levels of each analyzed gene in pepsin vs. control treated groups. (**C**) Graph depicts the mRNA ratios of *EGFR, IL6, IL1β*, *RELA(p65), mTOR, AKT1* and *BCL2,* in HCs exposed to pepsin at pH 7.0 or pH 6.0 relative to pepsin at pH 5.0. (Normalized mRNAs to *hGAPDH* reference control; mean ± standard error of two independent experiments). (*t*-test; multiple comparisons by Holm-Sidak, * *p* < 0.050; ** *p* < 0.005; *** *p* < 0.0005; **** *p* < 0.00005; ***** *p* < 0.000005; GraphPad Prism 7.0; means ± SD of three independent experiment).

Our gene expression analysis revealed that pepsin at pH 7.0 caused a significant upregulation of the analyzed genes, compared to pepsin at pH 5.0 and 6.0 (Figure 6A). As shown in Figure 6B and Table 1, HCs exposed to pepsin at pH 6.0 or 7.0 produced significantly higher transcriptional levels of *EGFR*, *IL6*, *IL1β*, *RELA(p65), mTOR, AKT1, TNF-α*, *STAT3* and *BCL2*, compared to pepsin at pH 5.0 and controls (Supplementary Material; Table S2). Figure 6C shows that *EGFR* was found to be the most upregulated gene between pepsin at pH 7.0 vs. pH 5.0 groups, followed by *IL6, IL1β*, *RELA(p65), mTOR, AKT1* and *BCL2*.

Pepsin at weakly acidic pH 5.0 also induced a significant upregulation of *TNF-α* and *IL6*, compared to controls. Pepsin did not affect mRNA levels of *PTGS2* and *PKI3CA*.

### 2.7. Correlations among Pepsin-Induced Protein and Gene Expression Levels

In order to estimate the correlation coefficient among the pepsin-induced protein levels of cytoplasmic p-EGFR, nuclear p-NF-*κ*B and p-STAT3, in the studied groups, we performed Spearman correlations. Spearman analysis revealed a significant positive correlation between cytoplasmic levels of p-EGFR and nuclear levels of p-NF-κB (*r* = 1, *p* = 0.0417, two-tailed), supporting possible interactions between EGFR and NF-κB under the effect of pepsin at variable pH points. Correlation analysis also showed a strong linear correlation between pepsin A and p-EGFR levels (*r* = 0.999207, *p* = 0.0001, two-tailed) (Supplementary Material; Figure S1C).

We also performed Spearman correlations, in order to estimate the correlation coefficient among pepsin-induced mRNA levels of the analyzed genes at variable pH. Our analysis revealed significant positive correlations between the pepsin-induced mRNA levels of (i) *EGFR* and *IL1β*, *mTOR,* or *RELA(p65)*; (ii) *RELA(p65)* and *IL1β*, or *mTOR* (iii) *IL1β* and *mTOR* (iv) *AKT1* and *IL6*, and (iv) *TNF-α* and *BCL2* (*r* = 1, *p* = 0.0417; two-tailed).

**Figure 7:**
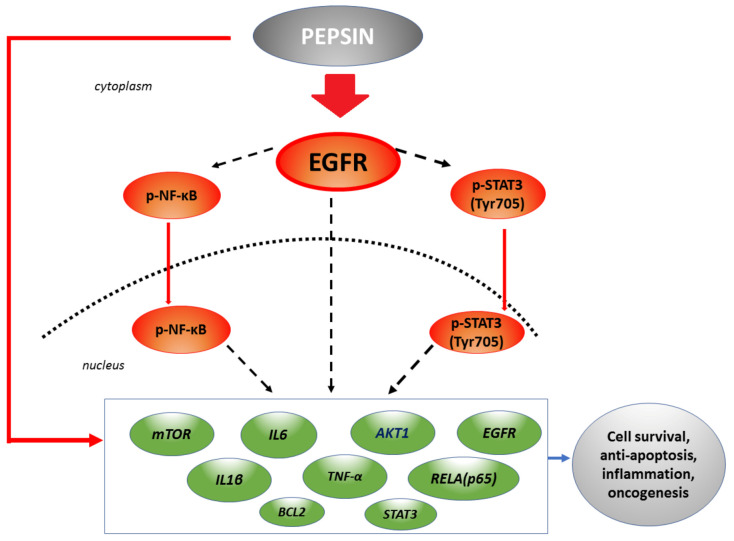
The effect of pepsin on epidermal growth factor receptor (EGFR) and its related signalling pathway in human hypopharyngeal primary cells, at less acidic LPR. Pepsin at slightly acidic or neutral pH causes significant overexpression of EGFR, accompanied by elevated activation of p-NF-*κ*B and p-STAT3, and a significant overexpression of EGFR-related oncogenic factors and cytokines, including *AKT1, mTOR*, *IL1β*, *IL6, TNF-α*, *STAT3* and *RELA(p65),* and antiapoptotic *BCL2*, previously linked to HNSCC [35,36,39,40,41,42,46,47,48,52]. (red arrows: pepsin effect in HCs; dashed black arrows: possible interactions; green boxes: genes upregulated by pepsin).

Finally, we used Spearman correlations analysis to estimate the correlation coefficient among pepsin-induced protein levels of EGFR, p-NF-*κ*B and p-STAT3, and mRNA levels of the analyzed genes, in the studied groups. Our analysis revealed significant positive correlations between (i) cytoplasmic p-EGFR and mRNA levels of *EGFR*, *IL1β*, *mTOR* or *RELA(p65)*, (ii) nuclear p-NF-*κ*B and mRNA levels of *RELA(p65), IL1β, IL6, EGFR* or *mTOR*, and (iii) nuclear p-STAT3 and mRNA levels of *AKT1* and *IL6* (*r* = 1, *p* = 0.0417; two-tailed). These correlations suggest possible interactions among these molecular events under the pepsin-effect (Figure 7).

## 3. Discussion

It has been recently proposed that non-acidic pepsin can be a significant contributor to laryngeal inflammation during a reflux episode [25]. Johnston et al. have shown in vitro that pepsin plays a role in the promotion of epithelial cell proliferation and carcinogenesis in the larynx and pharynx [6]. Notably, they showed that pepsin induced the overexpression of Ras protein, a downstream molecule of EGFR [6], while others have suggested that pepsin has a pro-tumorigenic effect produced through signaling pathways such as NF-*κ*B [61]. Although the importance of the pH in the development and promotion of malignancies of the upper aerodigestive tract remains unclear [62,63], recent studies document that similar to strongly acidic pH, less acidic pH can cause bile-reflux-related malignant transformation of hypopharyngeal epithelial cells and activation of NF-*κ*B and its anti-apoptotic molecular phenotype [64,65,66]. In our attempt to furtherly explore the pepsin-effect in vitro, we hypothesized that the pepsin-induced carcinogenesis is closely related to acidity by affecting specific oncogenic factors and signaling pathways. With regard to the range of acidified pepsin selected, test solutions below pH 5.0 were not selected as previously found to induce a pronounced cell death [22,23]. Thus, we chose pH 5.0 as the most acidic in this range, pH 6.0 since, according to Johnston et al., pepsin may not be completely inactive, and pH 7.0 where pepsin is inactive but remains stable for a long period of time [19]. Similarly, test solutions above pH 7.0 were omitted because pepsin above pH 7.0 is enzymatically inactive [67]. It is hoped that the data presented here contributes to clarifying the mechanism by which reflux may contribute to carcinogenesis, and thereby improve potential therapies to LPR-related carcinogenesis.

Previous explorations of the pepsin-effect in vitro clearly differ from our novel experimental approach. We performed repetitive exposure of pepsin to human hypopharyngeal primary cells instead of a single pepsin-treatment in cancer cells that have been previously investigated [21,61]. Other experimental investigations on the effect of pepsin included prolonged exposure to pepsin, even one to two hours [21,61,68]. We believe that the duration, as well as the intermittent in vitro exposure of pepsin is of great importance. Trying to be as close as possible to the clinical manifestations of a reflux episode, we treated HCs with pepsin for no more than 15 min at a time, once per day for a total of five days. This in vitro model allowed us to have a more realistic approach to the pepsin-effect.

Our novel experimental approach documented that a less acidic extracellular environment promotes the pepsin-oncogenic effect on HCs by causing specific molecular changes associated with activation of oncogenic EGFR and its related downstream signaling. Since no specific singling pathway for the effect of pepsin in vitro has been documented so far, we performed multiple assays including immunofluorescence, ELISA, western blot, quantitative RT-PCR and luciferase assay to confirm our findings. Therefore, our novel findings were confirmed from various angles and demonstrate the importance of pH levels in the overexpression of the above-mentioned markers.

Our findings demonstrated that pepsin, in vitro, at slightly acidic pH of 6.0 and in particular at neutral pH of 7.0 preserves cell survival and induces activation of specific oncogenic markers clinically associated with HNSCC [28,29,30,31,32,33,34,35,36,37,38,39,40,41,42,43,44,45,46,47,48,51,53]. Despite the possible pepsin-related stress toxicity and damage in cancer hypopharyngeal cells at either pH 7.4 or pH 7.0 [21,24], using our model, we provide evidence of the oncogenic effect of pepsin in normal HCs at pH 7.0 or 6.0, in line with other observations by Johnston’s team [6,25]. Elucidating the mechanism of the effect of pepsin on HCs, we showed the abundant activation of EGFR, which was documented by a significant overexpression of phospho-EGFR and *EGFR* mRNAs, accompanied by activation of two more oncogenic factors, NF-*κ*B and STAT3, and a significant upregulation of genes linked to related oncogenic signaling pathways, such as *EGFR/AKT1/mTOR*, *TNF-α/RELA(p65)/BCL2*, *STAT3* and cancer-related cytokines, *IL6* and *IL1β*. It is known that EGFR activation can exert an oncogenic effect by activating downstream oncogenic pathways, such as AKT/mTOR, STAT3 and NF-*κ*B [39,40,41,42,44], strongly supporting the role EGFR as a key molecule of the pepsin-induced tumorigenic effect on the hypopharynx in a less acidic environment.

The role of EGFR as a valuable prognostic and predictive biomarker in HNSCC has been widely established [39,69]. The relevance of EGFR pathway in HNSCC has led to the successful development of EGFR-targeted therapeutic approaches, including antibodies against EGFR, or downstream EGFR signaling inhibitors [70]. Understanding the mechanism of the effect of pepsin on LPR related inflammatory and neoplastic processes may help to derive sufficient prevention and successful therapeutic applications, including anti-EGFR antibodies and/or inhibitors of EGFR-related downstream signaling, including AKT1, mTOR, NF-*κ*B and STAT3 [44,45,71,72,73].

The role of NF-*κ*B in pepsin-related laryngopharyngeal neoplasia has been previously referred [29,30,51,74]. Previous studies supported a crosstalk between NF-*κ*B and EGFR-related down-stream pathways, such as AKT1 and STAT3 [45,46,47,48], to promote cell proliferation or antiapoptotic function. Our data from Western blot analysis, ELISA and immunofluorescence and luciferase assay support the contribution of NF-*κ*B to the effect of pepsin at slightly acidic or neutral pH, although its role may not be central.

The key role of STAT3 in head and neck carcinogenesis has been associated with the activation of several anti-apoptotic and oncogenic factors [31,44,75]. It has been previously shown that autophosphorylation of EGFR at Ty1092 recruits STAT3 [76]. Our findings support pepsin-induced EGFR/STAT3 interactions in HCs. However, our data could not exclude an EGFR independent activation of STAT3 [43] under the pepsin-effect which may be clarified by further exploration.

IL-6 is an inflammatory and head and neck cancer-related cytokine [35]. It has been shown that interleukin-6 (IL-6) can promote STAT3 activation in an autocrine manner [43,77]. Our data support that less acidic pepsin produces a significant overexpression of *IL6*, suggesting that transcriptional activation of *IL6* may be mediated by EGFR downstream signaling, including AKT1 and NF-*κ*B [42], thus contributing to STAT3 activation. The IL-1β is another inflammatory and cancer-related cytokine which has been recently found to affect EGFR transactivation in oral squamous cell carcinomas [36,78]. Our data showed that similarly to *IL6*, *IL1β* is highly overexpressed by the pepsin-effect at a slightly acidic or neutral pH compared to weekly acidic pH or controls, supporting a key role of *IL1β* as a pro-tumorigenic factor in this process [36].

One of the interesting findings regarding the pepsin-effect in various pH was obtained by the cell viability assay. Less acidic and neutral pH of pepsin preserved the cell survival when compared to more acidic pepsin (pH 5.0). These observations in combination with bcl-2 overexpression findings, from western blot and gene expression analyses, support the pepsin-antiapoptotic effect at less acidic pH.

Overall, our data give evidence that neutral environment induces the highest pepsin effect in treated HCs. Interestingly, at a more neutral extracellular environment, pepsin endocytosis appears to be more prominent [24]. Also, our findings indicate a possible positive effect of pepsin endocytosis on EGFR activation. However, the proportion of this effect and the precise mechanistic pathway or activation of EGFR and other upregulated oncogenic factors needs further exploration. The scheme in Figure 7, summarizes our findings that may propose a mechanism of the pepsin-effect at less acidic LPR, opening a window for future investigation using a mechanistic approach to explore EGFR, NF-*κ*B and STAT3 interactions in this process.

Current treatment of gastroesophageal reflux targeting the pH may improve patient’s symptoms [79,80]; however, our data strongly suggest this may not fully protect from pepsin internalization in epithelial cells of the upper aerodigestive tract and its tumorigenic effect [6,21,24,25]. Understanding the specific molecular mechanism by which less acidic pepsin containing refluxate exerts its effect in normal laryngopharyngeal cells, we may contribute not only to a more successful alternative treatment, but also to a more accurate diagnosis of early neoplastic events.

## 4. Materials and Methods

### 4.1. Cell Culture and Treatment Conditions

Human hypopharyngeal primary cells (HCs) from Celprogen Inc., Torrance, CA, USA were seeded in non-coated flasks and cultured in Human Hypopharyngeal Normal Cell Culture Media with Serum (Celprogen Inc., Torrance, CA, USA), at 37 °C in humidified air and 5% CO2. After reaching ∼80%, confluence medium was replaced with Serum Free Media (Celprogen Inc., Torrance, CA, USA) for 12 h before treatment.

**Figure 8:**
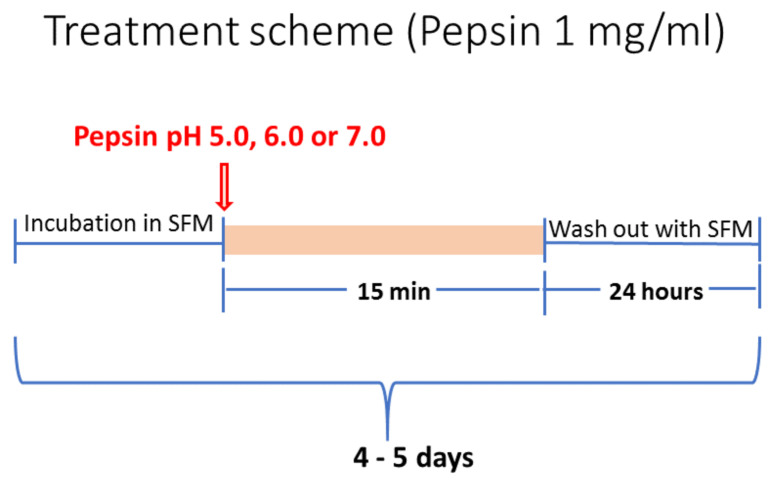
Schematic representation of pepsin-application in exposed HCs at variable pH (5.0, 6.0 and 7.0). (SFM: serum free medium).

HCs underwent a repetitive exposure to experimental and control media for 15 min, once per day, for 4–5 days (Figure 8). Experimental groups included (i) “Pepsin pH 5.0” (weakly acidic pepsin), (ii) “Pepsin pH 6.0” (slightly acidic pepsin) and (iii) “Pepsin pH 7.0” (neutral pepsin). All experimental groups were repetitively exposed to 1 mg/mL of pepsin (porcine pepsin; Sigma Aldrich, St. Louis, MO, USA) as previously described [59,81,82,83,84], in serum free medium (Dulbecco modified Eagle’s medium/F12, 1% pen/strep, Gibco^®^, Brooklyn, NY, USA). pH was adjusted by 1M HCl (using a pH meter). Pepsin at pH 4.0, the cut-off of reflux disease [85], was not used in the present study since pepsin and acid combination induces reduced cell survival in vitro [22,23,86]. HCs of control groups were treated with serum free medium, as used in experimental groups, at pH 7.0. Porcine pepsin (A) is commercially available and has similar activity to human pepsin (C) [87,88]. After each treatment we cultured the cells with serum free media until the next exposure cycle. Experimental and control groups were cultured in parallel. All the experiments were independently repeated three times. Cells were harvested immediately after the last treatment, by 0.05% trypsin-EDTA (Gibco^®^, Brooklyn, NY, USA).

### 4.2. Cell Viability Assay

Cell viability assay was performed to investigate if pepsin affects the cell survival at variable pH. We used Cell Titer-Glo^®^ Luminescent Cell Viability Assay (Promega Corp., Madison, WI, USA) to monitor the pepsin-effect at weakly acidic pH 5.0, slightly acidic pH 6.0, and neutral pH 7.0 on HCs treated cells, compared to controls. The cells were seeded at a density of 10,000 cells/well in 24-well plates. After 24 h the cells underwent a single exposure of pepsin at pH 5.0, 6.0, and 7.0 and treated-controls for 20 min. At the end of the treatment, the experimental and control media were removed from all groups and replaced by serum free media (Human Hypopharyngeal Normal Cell Culture Media Serum Free; Celprogen Inc., Torrance, CA, USA). Untreated controls were also grown in serum free media in parallel. Cells were cultured at 37 °C in humidified air and 5% CO_2_ for 5 days. Then luminescence was measured by a luminometer using Gen5 software (Synergy1, BIOTEK; Gen5^TM^ software, BioTek Instruments Inc., Winooski, VT, USA). All values were normalized to mean value of untreated controls. The statistically significant difference in cell viability was determined by *t*-test; multiple comparisons by Holm-Sidak and *p*-value < 0.05 (Graph Pad Prism 7.0, GraphPad Software Inc., San Diego, CA, USA). All experimental groups and controls were performed in triplicates.

### 4.3. Immunofluorescence Cell Staining

Immunofluorescence assay was performed, as previously described [54,57,58], to detect the pepsin-effect on activation of EGFR, STAT3, and NF-*κ*B, as well pepsin internalization, after pepsin exposure to HCs at variable pH relative to controls. Specifically, HCs were grown on multiwall chamber slides; Lab-Tek^®^ and treated with experimental media of 1 mg/mL of pepsin at pH 5.0, 6.0 or 7.0, and controls. Cells were fixed immediately after the final exposure to experimental or control media in 4% paraformaldehyde for 7 min, followed by 3 washes with PBS, permeabilization of cell membranes using 0.2% Triton X100-PBS (AmericanBio, Natick, MA, USA) for 3 min, and blocking with 2% bovine serum albumin (BSA)-PBS (Sigma-Aldrich, St. Louis, MO, USA) for 1 h. All groups were then incubated overnight at 4 C with 1:65 of primary antibodies for p-NF-*κ*B (rabbit polyclonal anti-phospho-p65 Ser536, AbD Serotec, BIO-RAD, CA, USA), p-STAT3 (Tyr705) (rabbit mAb, D3A7 XP^®^, Cell Signaling Technology, Inc., Danvers, MA, USA), p-EGFR (mouse monoclonal, Clone F-3; Santa Cruz Biotechnology Inc., Dallas, TX, USA), and Pepsin A (D-5) (mouse monoclonal, clone D-5, Santa Cruz Biotechnology Inc.; for the detection of Pepsin A and Pepsinogen A of human origin). Cells were washed by 1% Tween-PBS and incubated for 1 h, at room temperature, with 1:500 dilutions of secondary anti-rabbit or anti-mouse DyLight^®^488 (green; Vector Labs, Burlingame, CA, USA). Followingly, cells were mounted by Prolong Gold Mountant with diamidino-phenylindole (ProLong^®^ Diamond Antifade Mountant with DAPI; Life Technologies, Thermo Scientific, Franklin, MA, USA) for nuclear staining (blue color).

Zeiss Confocal microscope and Zen imaging software were used to examine stained slides and capture images, respectively (Zen imaging software, Carl Zeiss, microscopy GmbH, Jena, Germany), and expression levels were assessed by fluorescence intensity (mean ± SD bin count) from at least two independent images (>10 cells) (Zen imaging software), as previously described [56,89].

### 4.4. Luciferase Assay

Luciferase assay was performed to measure the transcriptional activity of the NF-*κ*B in HCs under pepsin exposure at each pH point compared to control. A NF-*κ*B reporter Vector [pGL4.32(luc2P/NF-κB-RE/Hygro)], a control vector [pGL4.27(luc2P/minP/Hygro)], a Lipofectamine^®^ 2000 (Invitrogen™, Thermo Fisher Scientific, Waltham, MA, USA) and a firefly Luciferase Assay system (Promega Corporation, Madison, WI, USA) were used, according to manufacturer’s procedure, as previously described [56,57,58]. The treatment was performed 24 h after transfection. The cells were treated with pepsin (1 mg/mL) at pH 5.0, 6.0 and 7.0 and controls, for 15 min and then media were replaced with serum-free medium. After 4–6 h incubation we measured luminescence using a luminometer and Gen5 software (Synergy1, BIOTEK; Gen5^TM^ software, BioTek Instruments Inc., Winooski, VT, USA). NF-*κ*B activity was expressed as ratios of mean values of NF-*κ*B reporter (NF-κBLuc2P) against mean values of control (Luc2P) for each condition (pepsin at pH 5.0, 6.0, 7.0 and control). Triplicate assays were performed for each treatment condition.

### 4.5. Protein Expression Analysis

Direct enzyme-linked immunosorbent assay (ELISA) and Western blot analysis were used to investigate the pepsin-effect on protein expression levels of NF-*κ*B, EGFR, STAT3, and Bcl-2, at variable pH.

#### 4.5.1. Enzyme-Linked Immunosorbent Assay Quantification

Direct enzyme-linked immunosorbent assay (ELISA) was performed to quantify the protein expression levels of cytoplasmic p-EGFR (Tyr 1092), and nuclear NF-*κ*B (p65) and p-STAT3 (Tyr 705), in experimental and control treated-HCs, as previously described and in Supplementary Materials [90].

Briefly, BCA-200 Protein Assay kit (Thermo Fisher Scientific, Waltham, MA, USA) was used to detect cytoplasmic and nuclear protein concentrations of all groups. Expression levels of p-EGFR, NF-*κ*B, and p-STAT3 were then quantified by ELISA. The primary antibodies in our assay were: p-EGFR (Clone F-3), NF-κB(p65) (Clone F-6), and p-STAT3 (clone B-7) mouse monoclonal antibodies HRP (Santa Cruz Biotechnology Inc., Dallas, TX, USA). Histone 1 (AE-4) and β-actin (Clone C4) (Santa Cruz Biotechnology Inc., Dallas, TX, USA) were used as a reference control for nuclear and cytoplasmic protein normalization, respectively). Absorbance values were measured by a microplate reader (Sunergy1, BIOTEK; Gen5^TM^ software, BioTek Instruments Inc., Winooski, VT, USA). Assays were carried out according to the manufacturer’s instructions, performed in triplicates and repeated two times, independently.

#### 4.5.2. Western Blot Analysis

Western blot analysis was used to determine the cytoplasmic or nuclear protein levels of p-EGFR (Tyr1092), bcl-2, p-NF-*κ*B (p65 S536), and p-STAT3(Tyr705), in experimental and control treated-HCs. Beta-actin and Histone 1 were used to normalize cytoplasmic and nuclear extracts, respectively, as previously described and in Supplementary Material [56,58]. We used 1:1000 primary antibodies for p-EGFR (Clone F-3), bcl2 (Clone N-19), p-NF-*κ*B (p65 Antibody 27.Ser 536), p-STAT3 (Tyr 705) (clone B-7), Histone 1 (AE-4) and β-actin (C4) (Santa Cruz Biotechnology Inc., Dallas, TX, USA). Protein levels were quantified by the Gel imaging system (Bio-Rad, Hercules, CA, USA) in each cytoplasmic or nuclear cellular compartment (Image Lab 5.2 analysis software, Bio-Rad, Hercules, CA, USA).

### 4.6. Quantitative Real-Time Polymerase Chain Reaction

Real-time polymerase chain reaction (qPCR) quantitative analysis was used to evaluate the transcriptional levels of *EGFR, IL1β, TNF-a, IL6, STAT3, mTOR, RELA(p65), BCL2, PKI3CA, AKT1, WNT5A,* and *PTGS2*. Total RNA was isolated from HCs exposed to pepsin at variable pH and controls (RNeasy mini kit; Qiagen Inc., Valencia, CA, USA). RNA quality and concentration were assessed by absorption ratios 260/280 nm (>2.0) and 260 nm, respectively (NanoDrop^TM^ 1000 spectrophotometer; Thermo Fisher Scientific, Waltham, MA, USA). Subsequently, reverse transcription from total RNA (iScript cDNA synthesis kit; Bio-Rad, Hercules, CA, USA) and qPCR analysis (Bio-Rad real-time thermal cycler CFX96^TM^; Bio-Rad, Hercules, CA, USA) were performed using specific primers for target genes and reference housekeeping gene, human glyceraldehyde 3-phosphate dehydrogenase (*h*GAPDH) (QuantiTect Primers Assays; Qiagen Inc., Valencia, CA, USA) (Supplementary Material; Table S1) and iQ^TM^ SYBR Green Supermix (Bio-Rad, Hercules, CA, USA). Our assay was performed on 96-well plates, in triplicates, and data were analyzed by CFX96^TM^ software [56,58]. Relative mRNA expression levels were estimated for each target gene compared to the reference control gene (ΔΔ*C*t).

### 4.7. Statistical Analysis

Multiple *t*-test analysis (GraphPad Prism 7 software; *t*-test; multiple comparisons by Holm-Sidak) was performed to demonstrate the differential expression (*p*-values) for each analyzed gene and protein expression between different experimental and control groups. Correlation analysis, by Spearman or Pearson, was performed to estimate the correlation coefficient between the transcriptional levels of the analyzed genes and the proteins in the different pH groups (*p*-values < 0.05).

## 5. Conclusions

We present for the first time a specific oncogenic pathway linked to a pepsin-related tumorigenic effect in hypopharyngeal cells in vitro. Our findings strongly support a central role of EGFR and its related signaling pathway in pepsin containing refluxate at slightly acidic and neutral pH, capable of promoting an oncogenic effect in hypopharyngeal cells. However, further exploration is required to clarify how pH affects pepsin-related cell proliferation rate in vitro and/or its oncogenic effect in vivo, and the role of EGFR in this process. We believe that our findings contribute both to the clarification of LPR pathophysiology and improvement of the current therapeutic approach in LPR-related carcinogenesis.

## Figures and Tables

**Table 1:** Pepsin-induced mRNA oncogenic phenotype in human hypopharyngeal primary cells (HCs).

	* Pepsin-Induced mRNA Phenotype in HCs		
	pH 5.0	pH 6.0	pH 7.0
*IL6*	↑2	↑158	↑285
*EGFR*	↓1.5	↑22	↑148
*IL1β*	↓1.2	↑4	↑46
*RELA*	↓2.1	↑7	↑8.6
*TNF-α*	↑2.9	↑8.3	↑6.6
*AKT1*	↑1.4	↑2.5	↑3.6
*mTOR*	1	↑2.8	↑3.5
*BCL-2*	↑1.3	↑3.1	↑2.7
*STAT3*	↑1.7	↑2	↑2
*Wnt5a*	↑1.8	↑1.5	↑1.2
*PTGS2*	1	1	↑1.2
*PKI3CA*	1	1	1

* Pepsin vs. control relative expression mRNA ratios (fold-changes). (mRNAs were normalized to *hGAPDH*); ↑, upregulation; ↓, downregulation; greyscale: genes were overexpressed in pepsin exposed HCs.

## Data Availability

Data is contained within the article or Supplementary Material.

## Supplementary Materials

The following are available online at https://www.mdpi.com/article/10.3390/ijms22084275/s1. Supplementary methods; Figure S1: Internalized-pepsin in human hypopharyngeal primary cells under exposure to weakly acidic pH 5.0, slightly acidic pH 6.0 and neutral pH 7.0, by immunofluorescence staining; Figure S2: Quantification of cytoplasmic p-EGFR(Tyr1092), nuclear p-STAT3 (Tyr705) and nuclear NF-κB (p65) levels, by ELISA, in human hypopharyngeal primary cells exposed to pepsin at variable pH and controls. Table S1: Human genes analyzed by real-time qPCR, in human hypopharyngeal primary cells; Table S2: Pepsin-induced upregulation of EGFR and its related pathways (A) Transcriptional levels of EGFR-related genes in pepsin-treated human hypopharyngeal primary cells, by real time qPCR. (B) Relative expression levels of EGFR-related genes in pepsin-treated human hypopharyngeal primary cells (pepsin at pH 5.0, 6.0 or 7.0 vs. control at pH 7.0).
