# Supplementary material for: Pepsin Promotes Activation of Epidermal Growth Factor Receptor and Downstream Oncogenic Pathways, at Slightly Acidic and Neutral pH, in Exposed Hypopharyngeal Cells
Source: Int J Mol Sci. 2021 Apr 20;22(8):4275. doi: 10.3390/ijms22084275 (PMC8074291; doi:10.3390/ijms22084275)
Supplement: Supplementary file 1 [file ijms-22-04275-s001.zip › ijms-1184605-supplementary.pdf]

Supplementary Material

Pepsin Promotes Activation of Epidermal Growth Factor Receptor and Downstream Pathways, at Slightly Acidic and Neutral pH, in Exposed Hypopharyngeal Cells

Supplementary methods

Enzyme-Linked Immunosorbent Assay for Total MSH2 Quantification: We performed a direct enzyme-linked immunosorbent assay (ELISA) to quantify cytoplasmic p-EGFR, and nuclear NF-kB, and p-STAT3 and expression levels. Nunc MaxiSorp™ 96-well plates (Invitrogen by Thermo Fisher Scientific) were coated with 100 μL of cytoplasmic and nuclear protein extracts from human hypopharyngeal primary cells (HCs), at a concentration of 10 μg/mL, in 1X coating buffer [protein added to coating buffer and mixed for 15 min; 1X coating buffer diluted from 5X stock (BUF030A; BIORAD) in dH_2O and mixed for 15 min]. The plates were covered and incubated at 4 °C overnight. The next day, the plates were washed 3 times in wash buffer (PBST; 0.05% v/v Tween-20 in PBS), incubated in 150 μL/well of blocking solution (1% w/v BSA in PBS) for 60 min at 37 °C, and then washed 4 times in wash buffer and incubated for 1 h at 37 °C, in 100 μL/well of primary p-EGFR (Clone F-3), p-STAT3 (clone B-7), NF-κB (Clone F-6), and β-actin (Clone C4; which was used as a reference control for cytoplasmic protein normalization) or Histone 1 (AE-4; which was used as a reference control for nuclear protein normalization) mouse monoclonal antibodies HRP (Santa Cruz Biotechnology). (We used 0.3 μg of each antibody per 1-2 μg of protein; antibodies were diluted in 1%BSA/PBS). Finally, the plates were washed 3 times in wash buffer and incubated in 100 μL/well of TMB Core+ substrate solution (3,3′, 5, 5′-tetramethylbenzidine plus hydrogen peroxide) BUF062C; Bio-Rad) for 30 min at room temperature in the dark. We read the absorbance values immediately at 600 nm using a microplate reader (Sunergy1, BIOTEK; Gen5™ software, BioTek Instruments Inc., Winooski, VT, USA). Protein standards for β-actin were used by 1:10 serial dilutions of a highly concentrated protein sample that was positive for p-EGFR. Assays were carried out according to the manufacturer's instructions and performed in triplicate. All experiments were repeated three times, independently.

Western blot analysis: Twenty to 30 μg of nuclear and cytoplasmic protein extracts from pepsin and control-treated HCs were heated at 70 °C for 10 min in sodium dodecyl sulfate-polyacrylamide gel electrophoresis Laemmli sample buffer (Bio-Rad, Hercules, CA, USA), and separated using 420–% Mini-PROTEAN TGX Tris/Glycine precast gels, at 150V for 1 h. We used precision plus pre-stained protein standards (Dual Color or Kaleidoscope, *BIO-RAD*) as molecular-weight size markers. Proteins were transferred onto a 0.45 mm nitrocellulose membrane, using a Trans-Blot Turbo transfer system (Bio-Rad). After blocking in 5% BSA, for 1 h, membranes were incubated with 1:1000 primary antibodies, p-EGFR (Clone F-3) and bcl2 (Clone N-19), p-NF-kB p65 Antibody (27.Ser 536), and 1:500 of p-STAT3 (Tyr 705) (clone B-7)(Santa Cruz Biotechnology Inc., Dallas, TX, USA), which were diluted in 5% BSA, overnight at 4 °C. Membranes were incubated for 1:30 h with goat anti-mouse horseradish peroxidase-conjugated secondary antibodies

(EMD Millipore, Burlington, MA, USA) at 1:5000, and chemiluminescence was determined using an enhanced chemiluminescence detection system (Clarity Western ECL Substrate, Bio-Rad). Membranes were also stripped using Restore™ Western Blot Stripping buffer (Pierce Biotechnology, Rockford, IL, USA) and reported with β-actin (C4; Santa Cruz Biotechnology Inc., Dallas, TX, USA) for cytoplasmic extracts and Histone 1 (AE-4; Santa Cruz Biotechnology Inc., Dallas, TX, USA) for nuclear extracts normalization. Protein levels were quantified by the Gel imaging system (Bio-Rad, Hercules, CA, USA) in each nuclear or cytoplasmic cellular compartment (Image Lab 5.2 analysis software, Bio-Rad, Hercules, CA, USA).

Supplementary Figure S1.

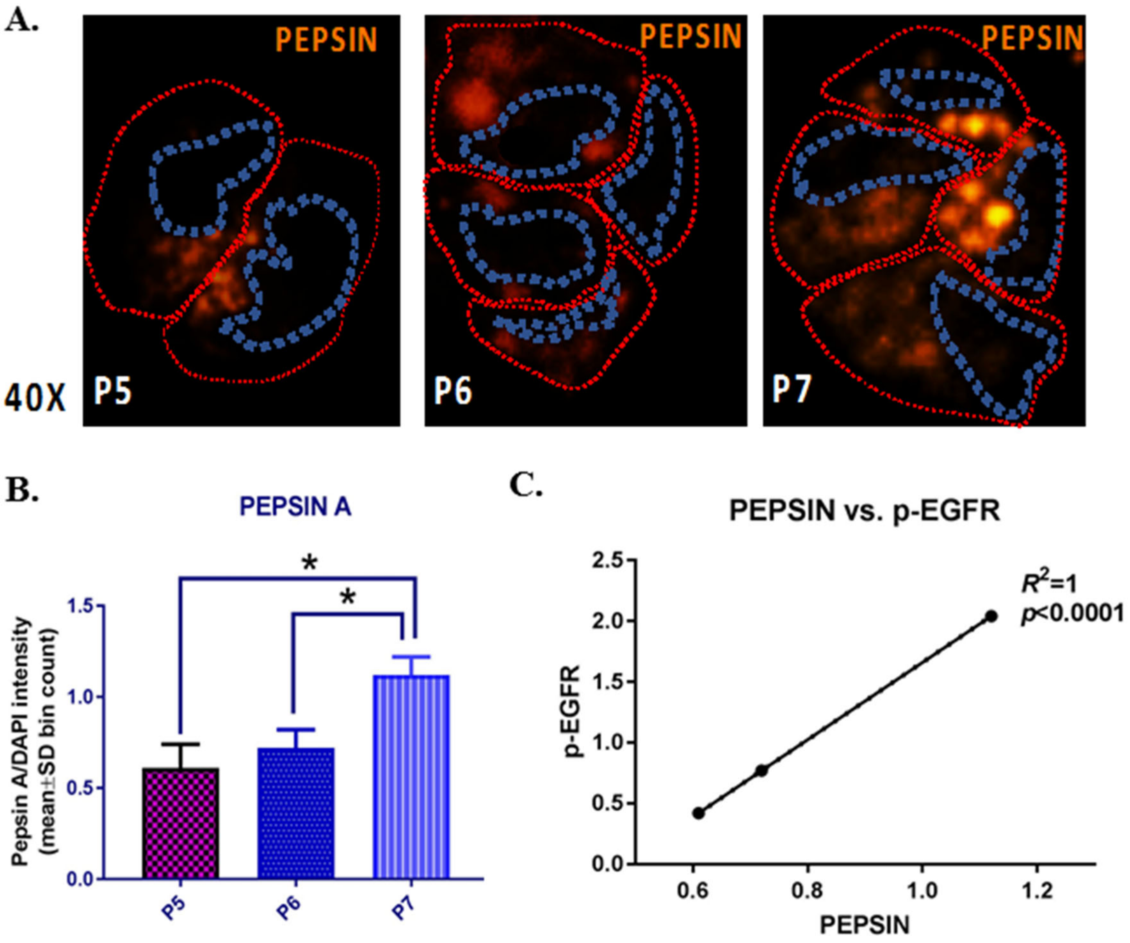

Supplementary Figure S1: Internalized-pepsin in human HCs under exposure at weakly acidic pH 5.0, slightly acidic pH 6.0 and neutral pH 7.0. **A.** Immunofluorescence staining for Pepsin A reveals that HCs exposed to pepsin at pH 7.0 (**P7**) presented the highest levels of internalized pepsin compared to slightly acidic pepsin at pH 6.0 (**P6**) or weakly acidic pepsin at pH 5.0 (**P5**). **B.** Columns of graph created by Graph Pad Prism 7.0 present the internalized-pepsin levels in HCs at pH 5.0, 6.0 and 7.0 (*$p<0.05$; t-test). **C.** Diagram presents the correlation coefficient between internalized pepsin A and p-EGFR (r=0.999207, p<0.0001; Graph Pad Prism 7.0; by Pearson)

(orange red: Pepsin A; red distinct line: cell membrane; blue distinct line: nuclear membrane: scale bar 20 ±μm by Zen imagining software).

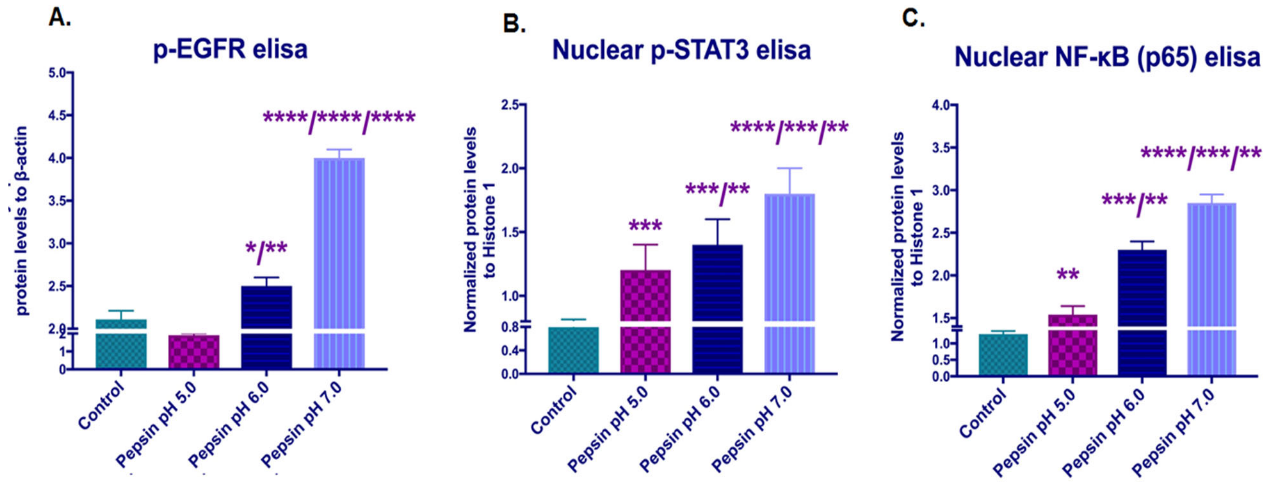

Supplementary Figure S2: Quantification of cytoplasmic p-EGFR(Tyr1092), and nuclear p-STAT3 (Tyr705) and NF-kB(p65), by ELISA, in HCs exposed to pepsin at variable pH and controls. Graphs created by GraphPad Prism 7.0 depict (**A**) cytoplasmic p-EGFR(Tyr1092), (**B**) nuclear p-STAT3(Tyr705), and (**C**) nuclear NF-kB(p65) in HCs exposed to controls at pH 7.0, and pepsin at pH 5.0, 6.0 and 7.0. (Controls: cells treated with media at pH 7.0 without pepsin; data obtained from three independent experiments; * $p < 0.05$; ** $p < 0.005$; *** $p < 0.0005$, ****$p < 0.00005$; by *t*-test; multiple comparisons by Holm-Sidak; GraphPad Prism 7.0; means±SD).

Supplementary Table S1: Human genes analyzed by real-time qPCR, in human hypopharyngeal cancer cells (HCs).

Gene	Detected transcripts	Amplicon length (bp)
***h*GAPDH**	NM_001256799,<br>NM_002046	95
BCL2	NM_000633	116
EGFR	NM_005228	80
RELA	NM_201282-4,<br>NM_001145138,<br>NM_001243984-5,<br>NM_021975	107
WNT5A	NM_001256105,<br>NM_003392	105
PKI3CA	NM_006218,<br>XM_006713658	108
TNFα	NM_000594	98
STAT3	NM_003150,<br>NM_139276	95
IL1β	NM_000576,<br>XM_006712496	117
IL6	NM_000600<br>XM_005249745	107
mTOR	NM_004958,<br>XM_005263438,<br>XM_005263439	66
AKT1	NM_001014431,<br>NM_001014432,<br>NM_005163,<br>XM_005267401	138
PTGS2	NM_000963	68

Supplementary Table S2. Pepsin-induced upregulation of EGFR and its related pathways.

A. Transcriptional levels of EGFR-related genes in pepsin-treated human HCs.

**Target gene/ *hGapdh** * ($\Delta\Delta^{CT}$)**	**Control pH 7.0**	**Pepsin pH 5.0**	**Pepsin pH 6.0**	**Pepsin pH 7.0**
IL6	1.000E-05	2.000E-05	1.580E-03	2.850E-03
EGFR	1.891E-06	1.237E-06	4.182E-05	2.800E-04
IL1β	4.805E-05	4.000E-05	1.900E-04	2.210E-03
RELA	5.100E-04	2.400E-04	3.570E-03	4.430E-03
TNF-α	3.500E-04	1.000E-03	2.930E-03	2.330E-03
AKT1	1.280E-03	1.800E-03	3.150E-03	4.620E-03
mTOR	6.318E-02	6.211E-02	1.795E-01	2.235E-01
BCL-2	4.320E-02	5.662E-02	1.352E-01	1.179E-01
STAT3	2.569E-01	4.264E-01	4.894E-01	4.954E-01
Wnt5a	2.000E-05	4.000E-05	3.000E-05	2.000E-05
PTGS2	6.076E-03	5.970E-03	6.350E-03	6.020E-03
PKI3CA	2.970E-01	3.093E-01	2.998E-01	3.409E-01

* normalization of mRNA levels using *hGapdh*

B. Relative expression levels of EGFR-related genes in pepsin-treated human HCs.

**Target gene/ *hGapdh** * ($\Delta\Delta^{CT}$)**	**Pepsin 5.0/ Cntl**	**Pepsin 6.0/ Cntl**	**Pepsin 7.0/ Cntl**
IL6	2.000E+00	1.580E+02	2.850E+02
EGFR	6.545E-01	2.212E+01	1.481E+02
IL1β	8.325E-01	3.954E+00	4.599E+01
RELA	4.706E-01	7.000E+00	8.686E+00
TNF-α	2.857E+00	8.371E+00	6.657E+00
AKT1	1.406E+00	2.461E+00	3.609E+00
mTOR	9.831E-01	2.841E+00	3.537E+00
BCL-2	1.311E+00	3.131E+00	2.730E+00
STAT3	1.660E+00	1.905E+00	1.928E+00
Wnt5a	2.000E+00	1.500E+00	1.000E+00
PTGS2	9.825E-01	1.045E+00	9.907E-01
PKI3CA	1.042E+00	1.009E+00	1.148E+00

* normalization of mRNA levels using *hGapdh;* Cntl: Control pH 7.0
